# Prevalence of overweight in adolescents from a Southern Brazilian city according to different anthropometric indexes

**DOI:** 10.1590/1984-0462/2021/39/2019277

**Published:** 2020-11-06

**Authors:** Augusto Gerhart Folmann, Vaneza Lira Waldow Wolf, Everton Paulo Roman, Gil Guerra-Júnior

**Affiliations:** a Universidade Estadual de Campinas, Campinas, SP, Brazil.; b Centro Universitário Fundação Assis Gurgacz, Cascavel, PR, Brazil.

**Keywords:** Obesity, Adolescents, Waist-to-height ratio, Obesidade, Adolescentes, Razão cintura-estatura

## Abstract

**Objective:**

To identify the prevalence of overweight in adolescents according to different classification criteria for obesity and somatic maturation stages.

**Methods:**

Cross-sectional study in 10 schools in a city from Southern Brazil, with 1715 adolescents. Height, weight, waist circumference, and neck circumference (NC) data were collected. Body Mass Index was classified according to World Health Organization (WHO) and Centers for Disease Control and Prevention criteria, and the waist-to-height ratio (WHtR) was classified according to Brazilian and European cut-off points. Somatic maturation was obtained through the Peak Height Velocity. The prevalence data were compared between sex and stages of somatic maturation; the concordance between different criteria was verified.

**Results:**

The prevalence of overweight was high in both sexes; WHO criteria showed that 34.5% of boys and 29.3% of girls were overweight. For the WHtR, the prevalence was 28.4% in boys and 23.7% in girls. NC classified 13.8% of boys and 15.8% of girls as being overweight. The prevalence of overweight was higher in adolescents before complete somatic maturation.

**Conclusions:**

The prevalence of overweight was high among adolescents. The boys presented higher frequency of overweight, except if NC was used to classify them. Adolescents before somatic maturation had a higher prevalence of overweight. NC showed a lower ability to track obese adolescents.

## RESUMO

**Objetivo:**

Identificar a prevalência de excesso de peso em adolescentes de acordo com diferentes critérios de classificação de obesidade e estágios de maturação somática.

**Métodos:**

Estudo transversal em dez escolas de um município da região Sul do Brasil, com 1.715 adolescentes. Dados de estatura, peso, circunferência da cintura e circunferência do pescoço (CP) foram coletados. O índice de massa corpórea (IMC) foi classificado com os critérios da Organização Mundial da Saúde (OMS) e do Centers for Disease Control and Prevention, e a razão cintura-estatura (RCE) foi classificada de acordo com pontos de corte brasileiros e europeus. A maturação somática foi obtida por meio do pico de velocidade do crescimento (PVC). Os dados de prevalência foram comparados entre os sexos e os estágios maturacionais; verificou-se a concordância entre os diferentes critérios.

**Resultados:**

A prevalência do excesso de peso foi elevada em ambos os sexos. Com o critério da OMS, a prevalência foi de 34,5% nos meninos e 29,3% nas meninas. Para a RCE, a prevalência foi de 28,4% nos meninos e 23,7% nas meninas. A CP rastreou 13,8% de excesso de peso nos meninos e 15,8% nas meninas. A prevalência de excesso de peso foi mais elevada em adolescentes antes da maturação somática completa.

**Conclusões:**

A prevalência do excesso de peso foi elevada entre os adolescentes. Os meninos apresentaram maior percentual de excesso de peso, exceto na variável CP. Adolescentes antes da maturação somática apresentaram maior prevalência de sobrepeso. A CP tem menor capacidade de rastrear adolescentes obesos.

## INTRODUCTION

A few decades ago, diet and physical activity patterns underwent major changes in developed and developing countries. In the 1980s, consumption of processed foods, instant foods and fast-food-style meals increased considerably.^1^ In addition, changes in leisure, mobility and work have reduced the practice of physical activity. Such transformations generated an “obesogenic” environment and led to major changes in body composition that culminated in an increase in obesity rates in the United States and Europe.^1^ ^,^ ^2^ However, obesity used to be a problem that was exclusive to developed countries, given that in underdeveloped countries, such as Brazil, the main problem was malnutrition.^3^ However, with the process of urbanization, and as underdeveloped countries have improved their economies, global influences have transformed the lifestyle of the population of these countries.^2^ As a result of the globalization process, refined and ultra-processed foods have become cheaper than organic foods, causing obesity rates to increase dramatically among the world population. Thus, the number of overweight people exceeded the number of malnourished people, and obesity has become a pandemic disease.^1^ ^,^ ^2^ ^,^ ^4^

Obesity is a complex disease resulting from the interaction between genetic propensity and various environmental factors,^4^ and it is characterized by an individual's excessive accumulation of body fat. Being overweight is an important risk factor for several comorbidities, such as cardiovascular disease, some types of cancer, type 2 diabetes, joint problems, in addition to psychosocial problems, such as poor quality of life, problems of social acceptance, depression and suicide.^4^ ^,^ ^5^ ^,^ ^6^ ^,^ ^7^ Obese children and adolescents are also at increased risk of cardiovascular problems and metabolic syndrome,^8^ ^,^ ^9^ in addition to being more likely to remain or become obese in adulthood.^10^

An estimate made in 2012 demonstrated that approximately 1.5 billion people are overweight, and that this number could reach 3.28 billion in 2030.^1^ Thus, periodically monitoring the prevalence of obesity is fundamental. There are several methods for assessing body composition in children and adolescents. Tools considered to be the gold standard for assessing body composition, such as dual-energy X-ray absorptiometry and computed tomography, are costly and limited in epidemiological studies. Body mass index (BMI) is the most non-invasive and accessible alternative and, therefore, is most often used. However, other methods, such as waist circumference (WC), neck circumference (NC) and waist-to-height ratio (WHtR), may be more effective in identifying risk factors related to obesity, such as diabetes, dyslipidemia, hypertension, among others.^11^ ^,^ ^12^ ^,^ ^13^ Thus, the aim of this study was to identify the prevalence of overweight in adolescents in a city in the southern region of Brazil, according to different criteria for the classification of obesity and different stages of somatic maturation.

## METHOD

This was a cross-sectional study carried out in ten schools, geographically distributed in the urban area of Cascavel, Paraná, southern Brazil. Of the ten schools, six were public and four were private. Sampling was carried out for convenience due to the availability and acceptance of schools invited to participate in the study. The research was carried out with 1,715 adolescents, of whom 840 were female and 875 were male. They were aged between 10 and 17 years old. Sixty-nine adolescents under or older than the age proposed by the study were not included in the final data analysis. During the research period, 24,292 students were enrolled in the public-school system and 4,384 in the private school system. No sample calculation was performed.

This study was conducted in accordance with the principles present in the Declaration of Helsinki and approved by the Research Ethics Committee of the Centro Universitário Fundação Assis Gurgacz - FAG (protocol no. 087/2013). Consent for data collection was obtained from the parents or guardians of the 1,715 students. Data were collected between September 2013 and August 2014 by a trained team, composed of academics from the Physical Education department at the Centro Universitário FAG.

Weight and height were collected according to the instructions in the Anthropometric Procedures Manual of the National Health and Nutrition Examination Survey*.* ^14^ Weight data were collected with a Tanita digital scale^®^ (Tanita Company, Tokyo, Japan), on a mass measurement scale presented in kilograms (kg). Height was assessed with a Seca^®^ wall stadiometer (*Seca*, Hamburg, Germany), with a scale from 0 to 200 centimeters (cm).

Nutritional status was classified based on different anthropometric criteria: BMI, WHtR and NC. The variables of weight and height were inserted in the formula BMI = weight (kg) ÷ height (m^2^ *)*. The BMI categorization was performed according to the cutoff points of the Centers for Disease Control and Prevention (CDC)^15^ and the World Health Organization (WHO).^16^ ^,^ ^17^ They are: low weight (1^st^ percentile to 5^th^ percentile), eutrophic (5^th^ percentile to 85^th^ percentile), overweight (85^th^ percentile to 95^th^ percentile) and obese (above the 95th percentile). The WHtR was obtained by dividing WC (cm) by height (cm) and subsequently classified according to the cutoff points established by Cintra et al.^18^ (overweight: ≥0.443 for girls and ≥0.439 for boys; obesity: ≥0.475 for girls and ≥0.489 for boys) and Sardinha et al.^12^ (overweight: ≥0.45 in males and ≥0.46 in females; obesity: ≥0.50 in males and ≥0.52 in females). WC was measured with a tape measure at the midpoint between the end of the iliac crest and the last rib, according to the procedures suggested by the CDC,^12^ and NC, with a flexible tape measure. The person being assessed stood and held their head upright, in line with the cricoid cartilage. NC data were compared by sex and age with the cutoff points suggested by Nafiu et al.^19^

The somatic maturation stage was obtained using the peak height velocity indicator (PHV), using the formula proposed by Moore et al: ^20^ PHV = -7.709133 + [0.0042232 x (age x height)], for females, and PHV = -7.999994 + [(0.0036124 x (age x height)], for males, with height values in centimeters and age in years. The data were classified into three groups: pre-PHV (-4 to -1), during PHV (-0.99 to 0.99) and post-PHV (1 to 4).

To verify the normality of the data, the Kolmogorov-Smirnov test was applied. The data were not normal. Initially, descriptive statistics were performed to obtain median values and 95% confidence intervals (95%CI). To compare the results between females and males, the Mann-Whitney U test was performed. To verify the difference between the different states of somatic maturation, the Kruskal-Wallis test was used. To verify the agreement between the different anthropometric assessment tools, the Kappa test was used. The level of agreement was classified as: there is no agreement (<0), minimum agreement (0-0.2), reasonable agreement (0.21-0.4), moderate agreement (0.41-0.6), substantial agreement (0.61- 0.8) and perfect agreement (0.81-1). The confidence level adopted was 95%. Data were analyzed using *statistical software Statistical Package for the Social Sciences* (SPSS) IBM^®^ (IBM*.*, Chicago, United States) version 20.0.

## RESULTS

The demographic characteristics of the 1,715 adolescents, separated by sex, are shown in Table 1. In males, 264 boys were in the pre-PHV stage, 334 were in the PHV stage and 277 were in the post-PHV stage; in females, 93 girls were in the pre-PHV stage, 266 were in the PHV stage and 481 were in the post-PHV stage. In general, for age, weight, height and NC, boys presented more data and more significant data than girls. However, the same did not have with regard to BMI. In both sexes, all variables were significantly greater in the more advanced stages of PHV compared to pre-PHV (Table 1). Table 2 shows the classification of nutritional status, based on different methods of anthropometric assessment.

**Table 1:** Demographic and anthropometric characteristics of 1715 (840 girls and 875 boys) adolescent students aged between 10 and 17 years old in a municipality in the Southern region of Brazil.

Variable	Sex	General Median (95%CI)	Pre-PHV	During PHV	Post-PHV	p-value¹
Age	Female	13.0 (13.2-13.4)	10.0 (10.1-10.3)	12.0 (11.8-12.1)	15,0 (14,6-14,8)	<0,001*(a ≠ b ≠ c) M/F
	Male	14.0 (13.3-13.6)	11.0 (11.1-11.3)	14.0 (13.4-13.5)	16,0 (15,5-15,7)	
	p-value^2^	<0.001 *				
Weight	Female	51.0 (51.3-52.9)	35.8 (36.4-39.7)	47.0 (46.8-49.3)	55,1 (56,1-58,1)	<0,001*(a ≠ b ≠ c) M/F
	Male	54.8 (54.9-57.0)	41.0 (42.3-45.1)	54.0 (55.0-57.7)	65,0 (65,6-68,8)	
	p-value^2^	<0.001*				
Height	Female	158.2 (156.9- 158.0)	143.8 (142.3-144.8)	154.3 (153.6-154.9)	161,0 (161,3-162,4)	<0,001*(a ≠ b ≠ c) M/F
	Male	164.5 (162.1-163.7)	149.0 (148.0-149.7)	165.0 (164.1-165.7)	174,0 (173,2-174,6)	
	p-value^2^	<0.001*				
BMI	Female	20.3 (20.6-21.1)	17.8 (17.7-19.0)	19.5 (19.6-20.5)	21,0 (21,4-22,0)	<0,001 (a ≠ b ≠ c) M/F
	Male	19.9 (20.5-21.0)	18.5 (19.0-20.0)	19.7 (20.1-21.0)	21,4 (21,7-22,6)	
	p-value^2^	>0.05				
NC	Female	29.7 (29.3-29.7)	27.0 (26.9-28.0)	29.0 (28.2-29.1)	30.5 (30.2-30.6)	<0,001*(a ≠ b ≠ c) M/F
	Male	31.5 (31.6-32.1)	29.0 (28.6-29.6)	32.0 (31.7-32.3)	34.0 (33.7-34.5)	
	p-value^2^	<0.001*				

PHV: peak height velocity; 95%CI: 95% confidence interval; BMI: body mass index; NC: neck circumference; M: male; F: female; *significant difference; ^1^Kruskal-Wallis test; ^2^Mann-Whitney U test.

**Table 2:** Classification of nutritional status by different assessment methods and criteria.

Variables		Sex	
		Male n (%)	Female n (%)
BMI WHO (2007)	Low weight	31 (3.5)	28 (3.3)
	Eutrophic	542 (61.9)	566 (67.4)
	Overweight	131 (15)	119 (14.2)
	Obese	171 (19.5)	127(15.1)
BMI CDC (2000)	Low weight	32 (3.7)	24 (2.9)
	Eutrophic	596 (68.1)	613 (73.1)
	Overweight	138 (15.8)	129 (15.4)
	Obese	109 (12.5)	73 (8.7)
WHtR (Sardine)	Eutrophic	673 (76.9)	695 (82.7)
	Overweight	123 (14.1)	109 (13.0)
	Obese	79 (9)	36 (4.3)
WHtR (Cintra)	Eutrophic	627 (71.7)	641 (76.3)
	Overweight	144 (16.5)	94 (11.2)
	Obese	104 (11.9)	105 (12.5)
NC	Eutrophic	754 (86.2)	707 (84.2)
	Overweight	121 (13.8)	133 (15.8)

BMI: Body Mass Index; WHO: World Health Organization; CDC Centers for Disease Control and Prevention; WHtR: waist-to-height ratio; NC: neck circumference.

The classification of nutritional status according to growth maturity is shown in Table 3. In females, 11.1% of the sample was in the pre-PHV stage, 31.7% was in the PHV state and 57.3% was in the post-PHV stage. Among males, 30.2% were in the pre-PHV stage, 38.2% were in the PHV stage and 31.7% were in the post-PHV stage. The group with the highest prevalence of overweight women was during PHV, with 33.9% of cases. In males, 42.1% of pre-PHV boys were overweight.

**Table 3:** Nutritional classification by stage of growth maturation.

Sex	Classification	Criterion	Growth maturation stage, n (%)			
			Pre-PHV	During PHV	Post-PHV	Total
Female (n = 840)	Low weight	CDC	4 (4.3)	11 (4.1)	10 (2.1)	25 (3.0)
		OMS	3 (3.2)	12 (4.5)	13 (2.7)	28 (3.3)
	Eutrophic	CDC	65 (69.9)	186 (69.9)	362 (75.3)	613 (73.0)
		OMS	61 (65.6)	164 (61.7)	341 (70.9)	566 (67.4)
	Overweight	CDC	13 (14.0)	42 (15.8)	74 (15.4)	129 (15.4)
		OMS	11 (11.8)	39 (14.7)	69 (14.3)	119 (14.2)
	Obese	CDC	11 (11.8)	27 (10.2)	35 (7.3)	73 (8.7)
		OMS	18 (19.4)	51 (19.2)	58 (12.1)	127 (15.1)
	Total		93 (11.1)	266 (31.7)	481 (57.3)	840 (100.0)
Male (n = 875)	Low weight	CDC	9 (3.4)	13 (3.9)	10 (3.6)	32 (3.7)
		OMS	8 (3.0)	13 (3.9)	10 (3.6)	31 (3.5)
	Eutrophic	CDC	165 (62.5)	237 (71.0)	194 (70.0)	596 (68.1)
		OMS	145 (54.9)	214 (64.1)	183 (66.1)	542 (61.9)
	Overweight	CDC	47 (17.8)	49 (14.7)	42 (15.2)	138 (15.8)
		OMS	39 (14.8)	48 (14.4)	44 (15.9)	131 (15.0)
	Obese	CDC	43 (16.3)	35 (10.5)	31 (11.2)	109 (12.5)
		OMS	72 (27.3)	59 (17.7)	40 (14.4)	171 (19.5)
	Total		264 (30.1)	334 (38.2)	277 (31.7)	875 (100.0)

PHV: peak height velocity; CDC: Centers for Disease Control and Prevention; WHO: World Health Organization

Table 4 shows the agreement between the different methods of anthropometric assessment. Although there is statistical significance, the BMI classifications (WHO and CDC) do not agree with the WHtR classifications (Sardinha and Cintra) or with the NC. The WHO’s BMI and CDC ratings are substantially in agreement (74.3%), as are the Sardinha and Cintra WhtR ratings (70.5%). The NC showed reasonable agreement with the WHtR classifications (27.3 and 21.4%), and did not show agreement with the BMI classifications.

**Table 4:** Agreement between the different methods of anthropometric assessment.

Criteria	BMI WHO		BMI CDC		WHtR (Sardinha)		WHtR (Cintra)		NC	
	Kappa	p-value	Kappa	p-value	Kappa	p-value	Kappa	p-value	Kappa	p-value
BMI WHO	1		0.743	<0.001	-0.087	<0.001	-0.066	<0,001	-0,070	<0,001
BMI CDC			1		-0.079	<0.001	-0.040	<0.001	-0,073	<0,001
WHtR (Sardinha)					1		0.705	<0.001	0.273	<0,001
WHtR (Cintra)							1		0.214	<0.001
NC									1	

BMI: Body Mass Index; WHO: World Health Organization; CDC: Centers for Disease Control and Prevention; WHtR: waist-to-height ratio; NC: neck circumference.

## DISCUSSION

The results found in this study with adolescents from 10 to 17 years of age point out significant differences in the variables weight, height, NC, WC and WHtR between males and females. The prevalence of being overweight varied according to the method used and the cutoff point. With the BMI and the WHtR, the prevalence of being overweight was high in both sexes. The NC, based on the cutoff points proposed by Nafiu et al.,^19^ showed a low prevalence of being overweight.

Adolescence is a stage of life in which major changes in body composition occur. In this study, this could be seen in the variables weight, height, BMI and NC, which showed significant differences between the somatic maturation groups. In males, being overweight was higher in adolescents who had not yet reached PHV, and in females, overweight and obesity rates were higher in adolescents during PHV.

The results of this study were similar to other prevalence studies carried out in Brazil. In the study of cardiovascular risks in adolescents (ERICA),^21^ which used the WHO cutoff points as a criterion, approximately 30.1% of female adolescents in the southern region of Brazil were overweight, while 29.4% of the boys of the same stage were overweight. Another study carried out in Acre by Farias et al.^22^ that used the cutoff points of the CDC, found results similar to this research: 30% of boys and 24.2% of girls were overweight. The prevalence of being overweight in adolescents in the city of Fortaleza^23^ was also similar: 33.7% of boys, of higher social class, and 24.8% of girls. A study by Cintra et al.,^18^ conducted in the city of São Paulo, found that 31.6% of boys and 25.4% of girls were overweight.

In South America, being overweight affects more than 50% of the adult population.^24^ The prevalence data among adolescents from countries such as Argentina (29.1% in males and 23.6% in females) and Paraguay (21.3% in boys and 24.3% in girls)^24^ were similar to the data in this study. Uruguay (31.2% males and 37.7% females) and Chile (37.0% boys and 31.6% girls)^24^ have higher rates of being overweight in relation to those in this study.

BMI is the tool that is most used to define overweight and obesity, both in adults, and in children and adolescents.^25^ However, despite its popularity and ease of use, its ability to detect regional obesity and to predict risk factors is weaker than other tools, such as NC and WHtR.^11^ ^,^ ^12^ ^,^ ^13^ ^,^ ^26^ ^,^ ^27^ The median of the WHtR presented in this study by adolescents of both sexes was lower than that found in the studies by Sardinha et al.^12^ (0.44 for boys and 0.44 for girls) and by Cintra et al.^18^ (0.45 for boys and 0.44 for girls). However, the adolescents in this study had a higher prevalence of being overweight than those in the study by Cintra et al.^18^ (20.9% of boys and 18.5% of girls).

It should be noted that another inexpensive, easy to obtain and reliable method is NC.^19^ ^,^ ^28^ The median NC in this study was similar to the values presented in the study by Nafiu et al.^19^ (30.9 cm in females and 33.1 cm in males, in adolescents with low BMI) and de Silva et al.,^28^ who assessed prepubescent and pubescent adolescents (30.6 cm in prepubescent girls and 32.6 cm in pubescent girls; and 32.8 cm in prepubescent boys and 35.4 cm in pubescent boys). Despite tracking a lower percentage of overweight and obesity, NC has a greater capacity to identify risks of diseases resulting from obesity, such as hypertension,^29^ dyslipidemia, insulin resistance and other metabolic complications.^30^ Its predictive ability is explained by the power to identify the distribution of body fat. In children and adolescents, the distribution of fat is more harmful to health than just excess fat.^31^ NC is a tool that assesses the distribution of subcutaneous fat in the upper body, the region that most secretes free fatty acids in the systemic circulation.^31^ Another strength of NC is its convenience and accuracy. It has good inter- and intra-rater reliability,^32^ in addition it does not require children to remove their clothes. Furthermore NC also does not require a scale or stadiometer. ^30^ ^,^ ^31^

The median age of PHV was 14 years old (95%CI 13.44-13.59) in males and 12 years old (95%CI 11.87-12.07) in females. In males, 30.2% of the sample had not yet reached PHV, compared to 11.1% of females. The data in this study indicate that being overweight was higher in adolescents, of both sexes, before their complete somatic maturity. In males, the prevalence of being overweight was higher in the pre-PHV stage (42.1%), and in females, in the stage during PHV (31.2%). Adolescence is a period of intense changes in body composition: height, weight, BMI, fat, lean mass and bone mineral content increase during maturation.^33^ The higher values of excess body weight before somatic maturation can be explained by the increase in body mass and the accumulation of fat necessary for the pubertal spurt to occur.^33^ ^,^ ^34^ For this reason, monitoring body composition during puberty is extremely important.

Adipose tissue is a major component of body composition and is directly involved with hormonal interactions. Thus, obesity is involved with growth and maturation factors during puberty that affect aspects of metabolic programming, such as energy expenditure and insulin resistance.^33^ Furthermore, being overweight is also related to early maturation, including decreasing age at menarche,^35^ in addition to having a mediating role in the relationship between biological maturation and metabolic risks.^36^

The criteria for classification of nutritional status using BMI (WHO and CDC) showed substantial agreement by the Kappa test. The same occurred among the WHtR criteria proposed by Cintra et al.^18^ and Sardinha et al.^12^ However, there was no agreement when different methods were compared.

Undoubtedly, obesity is one of the most serious chronic diseases that the world has been facing and is identified a potential cause for the decline in life expectancy in the 21st century.^37^ Its development in the early ages is known to be a major problem. WHO member countries, concerned with the risks of increasing the prevalence of obesity, have set a goal to contain the increase in obesity rates by 2025.^24^ Thus, monitoring the prevalence of obesity is essential for the planning of prevention strategies against the increase in the rate of overweight adolescents, making it important that this type of monitoring is carried out with different tools.

We must mention some limitations in this study and consider them in the interpretation and extrapolation of the results. Sampling was carried out for convenience and the data were obtained through a cross-sectional study. Gold standard methods were not used to assess body composition. Other information, such as how these adolescents spend their free time, the hours they spend in front of electronics, their levels of physical activity, their daily sleep hours, the history of obesity within their family and daily caloric intake, were not analyzed. The data were collected in a specific region of the country and, therefore, it is suggested that we compare these results with other regions.

Our research sought to assess the prevalence of being overweight and obese based on different methods of classifying obesity in adolescents. A high rate of being overweight and central overweight/obesity was found among adolescents in the western region of the state of Paraná, pointing to the need to adopt strategies to change the nutritional habits of this specific population. With that, we suggest conducting further studies to investigate the association of obesity with risk factors in the region.

The present study concluded that the prevalence of being overweight was high among adolescents participating in the research. The percentage of overweight male adolescents was higher than that of females in most nutritional status classification criteria. Somatic premature adolescents had a higher prevalence of being overweight. The study suggests that, for BMI, WHO cutoff points can track a higher percentage of obese adolescents; among the WHtR, the one with the highest rates of being overweight was the criterion proposed by Cintra et al.^18^ NC has been shown to have less ability to track overweight or obese adolescents.

## References

[1] Popkin BM, Adair LS, Ng SW (2012). Global nutrition transition and the pandemic of obesity in
developing countries. Nutr Rev.

[2] Gluckman PD, Hanson M, Zimmet P, Forrester T (2011). Losing the war against obesity: the need for a developmental
perspective. Sci Transl Med.

[3] Monteiro CA, Benicio MH, Konno SC, Silva AC, Lima AL, Conde WL (2009). Causes for the decline in child undernutrition in Brazil,
1996-2007. Rev Saude Publica.

[4] Roth J, Qiang X, Marban SL, Redelt H, Lowell BC (2004). The obesity pandemic: where have we been and where are we
going?. Obes Res.

[5] Yan LL, Daviglus ML, Liu K, Pirzada A, Garside DB, Schiffer L (2004). BMI and health-related quality of life in adults 65 years and
older. Obes Res.

[6] Dong C, Li W, Li D, Price RA (2006). Extreme obesity is associated with attempted suicides: results
from a family study. Int J Epidemiol.

[7] Noh J, Kwon YD, Park J, Kim J (2015). Body mass index and depressive symptoms in middle aged and older
adults. BMC Public Health.

[8] Sun SS, Grave GD, Siervogel RM, Pickoff AA, Arslanian SS, Daniels SR (2007). Systolic blood pressure in childhood predicts hypertension and
metabolic syndrome later in life. Pediatrics.

[9] Sun SS, Liang R, Huang TT, Daniels SR, Arslanian SS, Liu K (2008). Childhood obesity predicts adult metabolic syndrome: the fels
longitudinal study. J Pediatr.

[10] Guo SS, Wu W, Chumlea W, Roche A (2002). Predicting overweight and obesity in adulthood from body mass
index values in childhood and adolescence. Am J Clin Nutr.

[11] Ashwell M, Gunn P, Gibson S (2012). Waist-to-height ratio is a better screening tool than waist
circumference and BMI for adult cardiometabolic risk factors: systematic
review and meta-analysis. Obes Rev.

[12] Sardinha LB, Santos DA, Silva AM, Grøntved A, Andersen LB, Ekelund U (2016). A comparison between BMI, waist circumference, and
waist-to-height ratio for identifying cardio-metabolic risk in children and
adolescents. PLoS One.

[13] Janssen I, Katzmarzyk PT, Ross R (2004). Waist circumference and not body mass index explains
obesity-related health risk. Am J Clin Nutr.

[14] Centres for Disease Control and Prevention (2007). National Health and Nutrition Examination Survey (NHANES): anthropometry
procedures manual.

[15] Centres for Disease Control and Prevention (2001). Data table of BMI-for-age charts.

[16] World Health Organization (2007). BMI-for-age girls.

[17] World Health Organization (2007). BMI-for-age boys.

[18] de Pádua Cintra I, Zanetti Passos MA, dos Santos LC, da Costa Machado C, Fisberg M (2014). Waist-to-height ratio percentiles and cutoffs for obesity: a
cross-sectional study in Brazilian adolescents. J Health Popul Nutr.

[19] Nafiu OO, Burke C, Lee J, Voepel-Lewis T, Malviya S, Tremper KK (2010). Neck circumference as a screening measure for identifying
children with high body mass index. Pediatrics.

[20] Moore SA, Mckay HA, Macdonald H, Nettlefold L, Baxter-Jones AD, Cameron L (2015). Enhancing a somatic maturity prediction model. Med Sci Sports Exerc.

[21] Bloch KV, Klein CH, Szklo M, Kuschnir MC, Abreu G, Barufaldi LA (2016). ERICA: Prevalences of hypertension and obesity in Brazilian
adolescents. Rev Saude Publica.

[22] Moreira MA, Cabral PC, Ferreira HS, Lira PI (2012). Overweight and associated factors in children from northeasten
Brazil. J Pediatr (Rio J).

[23] Campos LD, Leite AJ, Almeida PC (2006). Socioeconomic status and its influence on the prevalence of
overweight and obesity among adolescent school children in the city of
Fortaleza, Brazil. Rev Nutr.

[24] Ng M, Fleming T, Robinson M, Thomson B, Graetz N, Margono C (2014). Global, regional, and national prevalence of overweight and
obesity in children and adults during 1980-2013: a systematic analysis for
the Global Burden of Disease Study 2013. Lancet.

[25] World Health Organization (2000). Obesity: preventing and managing the global epidemic.

[26] Haun DR, Pitanga FJ, Lessa I (2009). Waist-height ratio compared to other anthropometric indicators of
obesity as predictors of high coronary risk. Rev Assoc Med Bras (1992).

[27] Lee CM, Huxley RR, Wildman RP, Woodward M (2008). Indices of abdominal obesity are better discriminators of
cardiovascular risk factors than BMI: a meta-analysis. J Clin Epidemiol.

[28] Silva CC, Zambon MP, Vasques AC, Rodrigues AM, Camilo DF, Antonio MA (2014). Neck circumference as a new anthropometric indicator for
prediction of insulin resistance and components of metabolic syndrome in
adolescents: Brazilian Metabolic Syndrome Study. Rev Paul Pediatr.

[29] Nafiu OO, Zepeda A, Curcio C, Prasad Y (2014). Association of neck circumference and obesity status with
elevated blood pressure in children. J Hum Hypertens.

[30] Ma C, Wang R, Liu Y, Lu Q, Liu X, Yin F (2017). Diagnostic performance of neck circumference to identify
overweight and obesity as defined by body mass index in children and
adolescents: systematic review and meta-analysis. Ann Hum Biol.

[31] Jensen MD (2008). Role of body fat distribution and the metabolic complications of
obesity. J Clin Endocrinol Metab.

[32] Laberge RC, Vaccani JP, Gow RM, Gaboury I, Hoey L, Katz SL (2009). Inter- and Intra-rater reliability of neck circumference
measurements in children. Pediatr Pulmonol.

[33] Siervogel RM, Demerath EW, Schubert C, Remsberg KE, Chumlea WC, Sun S (2003). Puberty and body composition. Horm Res.

[34] Barbosa KB, Franceschini SC, Priore SE (2006). Influence of the stages of sexual maturation in the nutritional
status, anthropometrics and corporal composition of
adolescents. Rev Bras Saude Matern Infant.

[35] Kaplowitz PB (2008). Link between body fat and the timing of puberty. Pediatrics.

[36] Werneck AO, Silva DR, Collings PJ, Fernandes RA, Ronque ER, Barbosa DS (2016). Biological maturation, central adiposity, and metabolic risk in
adolescents: a mediation analysis. Child Obes.

[37] Olshansky SJ, Passaro DJ, Hershow RC, Layden J, Carnes BA, Brody J (2005). A potential decline in life expectancy in the United States in
the 21st century. N Engl J Med.

